# OpenCL-accelerated first-principles calculations of all-electron quantum perturbations on HPC resources

**DOI:** 10.3389/fchem.2023.1156891

**Published:** 2023-05-26

**Authors:** Zhikun Wu, Honghui Shang, Yangjun Wu, Zhongcheng Zhang, Ying Liu, Yuyang Zhang, Yucheng Ouyang, Huimin Cui, Xiaobing Feng

**Affiliations:** Institute of Computing Technology, Chinese Academy of Sciences, Beijing, China

**Keywords:** OpenCL, DFPT, GPU, optimization, heterogeneous

## Abstract

We have proposed, for the first time, an OpenCL implementation for the all-electron density-functional perturbation theory (DFPT) calculations in FHI-aims, which can effectively compute all its time-consuming simulation stages, i.e., the real-space integration of the response density, the Poisson solver for the calculation of the electrostatic potential, and the response Hamiltonian matrix, by utilizing various heterogeneous accelerators. Furthermore, to fully exploit the massively parallel computing capabilities, we have performed a series of general-purpose graphics processing unit (GPGPU)-targeted optimizations that significantly improved the execution efficiency by reducing register requirements, branch divergence, and memory transactions. Evaluations on the Sugon supercomputer have shown that notable speedups can be achieved across various materials.

## 1 Introduction

Density-functional perturbation theory (DFPT) allows for the study of a large variety of physical observables; for example, it can solve the physical response properties of Raman strength, polarization, and dielectric constants with high precision. The theory is widely used in molecular and materials simulations (Baroni et al., 1987a; 1987b; de Gironcoli et al., 1989; de Gironcoli, 1995; Giannozzi et al., 1991; Gonze, 1997; Gonze and Lee, 1997).

FHI-aims is an all-electron electronic structure code based on numerical atom-centered orbitals (Blum et al., 2009). It enables first-principle simulations with very high numerical accuracy for production calculations and is a popular implementation of DFPT (Shang et al., 2017; 2018). Recently, the DFPT part of FHI-aims has been implemented on the new generation Sunway supercomputer, which increases the simulation scale by several orders of magnitude (Shang et al., 2021). In particular, all of its time-consuming simulation stages have been offloaded to the heterogeneous many-core accelerators and accelerated by a set of optimizations targeting the Sunway architecture, such as utilizing the DMA mechanism and exploiting SIMD shuffling. At the same time, heterogeneous accelerators have been the focus of more and more scientific simulations for their massive computational capabilities. However, to the best of our knowledge, FHI-aims has not yet been implemented and accelerated on more general heterogeneous architectures, such as general-purpose graphics processing units (GPGPUs), making it difficult for scientists to leverage powerful computing capabilities on modern supercomputers.

In this paper, we proposed an OpenCL implementation of FHI-aims, together with efficient optimizations targeting GPGPUs. This implementation makes the following contributions:

• An OpenCL implementation of FHI-aims. The three time-consuming stages of FHI-aims, i.e., real-space integration of the response density, Poisson solver for the electrostatic potential, and calculation of the response Hamiltonian matrix, were implemented in OpenCL, allowing the end-to-end simulation with FHI-aims to be accelerated across different accelerators. As a result, FHI-aims could be utilized more extensively for more scientific findings.
• Fine-grained parallelism exploited for GPGPUs. Radial spherical grid points centered on the geometric coordinates of the nucleus were processed in fine-grained parallelism; that is, several grid points were mapped to one thread rather than several batches (typically including hundreds to thousands of grid points) being mapped to one thread on Sunway. The explicitly expressed fine-grained parallelism provided abundant parallelism for GPGPUs, allowing FHI-aims to fully utilize their massively parallel computing capabilities.
• Efficient data placement strategy targeting GPGPUs. Intermediate computing results, such as relevant numbers of a density matrix, were placed into different memory regions according to their access patterns to fully exploit the complex memory hierarchy on GPGPUs. Data placement strategies were determined to minimize data movement across different memory regions under the capacity constraint of each memory region. In particular, data were placed into optimal storage on GPGPUs, including the register files, on-chip SPM, and off-chip memory that can be cached.
• Highly convergent control flow designed for GPGPUs. Existing branches in FHI-aims, for example, branches caused by different cases of Fp-functions for periodic systems, were statically or dynamically eliminated to avoid useless computations on GPGPUs. In particular, we statically resolved some control flows in OpenCL kernels by synthesizing information from the OpenCL host codes and hoisting it to CPU to prevent GPGPUs from executing divergent control flows. Furthermore, we dynamically resolved some control flows by passing information collected during the execution of OpenCL host codes to the kernel to effectively eliminate branch divergence.
• Experimental results. We evaluated our optimized OpenCL implementation of FHI-aims on the Sugon supercomputer, and the results indicate that the performances of all three time-consuming stages improved by up to 5.3× on a Sugon node.

## 2 Background

This section introduces the DFPT method, the OpenCL programming framework, and the Hygon GPU architecture.

### 2.1 The density-functional perturbation

The quantum response/perturbation theory is the way to obtain the physical properties of the system that can be calculated within the uniform quantum mechanical framework by means of density-functional perturbation theory (DFPT) (Baroni et al., 1987a; 1987b; de Gironcoli et al., 1989; de Gironcoli, 1995; Giannozzi et al., 1991; Gonze, 1997; Gonze and Lee, 1997; Baroni et al., 2001).

To theoretically determine the properties mentioned above, numerical solutions for the quantum perturbation form of the Schrödinger equation are required. Initially, a single-particle approximation is employed to simplify the complex many-body problem. Subsequently, the single-particle wave functions are represented as a linear combination of predetermined basis functions. This allows us to formulate a matrix equation that can be solved numerically. The specific numerical method chosen depends on the form of the basis function, resulting in varying outcomes.

Various types of basis sets can be utilized in different computational codes. These basis sets can include plane-waves [Quantum ESPRESSO (Giannozzi et al., 2009), VASP (Kresse and Hafner, 1993), and QBox (Gygi, 2008)], uniform real-space grids [Octopus (Andrade et al., 2015)], periodic sinc functions [ONETEP (Skylaris et al., 2005)], b-spline functions [CONQUEST (Bowler et al., 2006)], finite elements [DFT-FE (Das et al., 2019)], and wavelets [BigDFT (Mohr et al., 2014)]. Although these basis sets can be systematically converged, the computation required to accurately represent the oscillatory behavior near the atomic nucleus can be prohibitively heavy. For instance, 10^5^ plane waves may be necessary to represent one core orbital.

As a result, when using the above basis sets, the pseudization methods (Lejaeghere et al., 2016) using pseudo-potentials or projector-augmented wave (PAW) have been introduced, in which the core potential has been replaced with a ‘fake’ one. Although the pseudo-potentials have been carefully constructed to keep the valence part to be consistent with the all-electron method, the information of the core shells is still missing. In order to consider the core and valence states on the equal footing, the all-electron approaches have been developed, e.g. linearized augmented plane wave (LAPW) (Madsen et al., 2001), linear muffin-tin orbital (LMTO) (Methfessel et al., 1989) methods and all-electron numerical atomic orbitals method (Delley, 1990; Blum et al., 2009)). Such all-electron methods can achieve better precision compared with the pseudization method (Lejaeghere et al., 2016).

In this work, the all-electron approach with the numerical atomic basis functions was used to achieve high-precision results, especially for the prediction of physical properties with the DFPT method. In this scheme, the all-electron atomic orbitals were discretized using the atom-centered grid (Becke, 1988) to treat the all-electron full-potential systems where the integrand is dominated by cusps at the atomic nuclei. In fact, the pseudopotential-based method could affect the results for the materials containing *d* electrons because the nonlinear core corrections in the pseudopotentials could influence the final high-frequency dielectric constants. For example, the difference between with and without nonlinear core corrections in the dielectric constant calculation of gallium antimonide (GaSb) is approximately 6%. Such nonlinear core correction pseudopotentials must be adopted to obtain the correct values. Our all-electron value (16.0) (Shang et al., 2018) is in good agreement with that of Dal Corso *et al.* (16.7), which adopted such nonlinear core corrections pseudopotentials. We found that the significantly larger value of Giannozzi et al. (18.1) was related to a smaller k-point grid used in their calculation, and no nonlinear core corrections were considered in their pseudopotentials.

The DFPT approach was implemented in a few computational packages, such as Quantum ESPRESSO (Giannozzi et al., 2009), Crystal (Maschio et al., 2012), and FHI-aims (Shang et al., 2017). To the best of our knowledge, their maximum parallel scale is thousands of cores on x86 platforms.

For codes using localized atomic orbitals, such as a Gaussian basis set, the DFPT was mainly implemented to treat finite, isolated systems (Pople et al., 1979; Frisch et al., 1990). Only a few literature reports exist on the treatment of periodic boundary conditions with such basis sets (Izmaylov and Scuseria, 2007), with only the perturbations corresponding to the unit cell (Γ-point perturbations). In fact, for periodic systems, the linear algebra operations are different between DFT and DFPT for the following two reasons: 1) the DFT calculations involve eigenvalue solving problems (AX = bX), whereas the DFPT calculations solve linear equations (AX = B). 2) Because the perturbations in DFPT destroy the boundary conditions of periodic systems and the atomic displacements cause a change in the entire basis set, the construction of the related matrix elements is complex and not as straightforward as expected. Therefore, compared to DFT, the parallelization and the corresponding optimizations are much more complicated.

Due to the complexity of the DFPT formula, the implementations of DFPT using an all-electrons scheme for both the finite (molecules) and extended (periodic) systems are rare and lack subsequent optimization; for example, the implementations using linear muffin-tin orbitals (Savrasov and Savrasov, 1996), linearized augmented plane waves (Yu and Krakauer, 1994; Kouba et al., 2001), or a Gaussian basis set for only the electric field perturbation (Maschio et al., 2012) have not been reported to scale to massive MPI processes.

The first-principles perturbation calculation is key to determining the response’s physical properties. Here, we only briefly summarized the first-principles quantum perturbation approach. Throughout the text, we used spin-unpolarized notation for the sake of simplicity, but a formal generalization to collinear (scalar) spin treatment is straightforward. In the following chapters, we will use subscripts *i*, *j* for occupied KS orbitals; *a* for the corresponding unoccupied (virtual) KS orbitals; *p*, *q* for the entire set of KS orbitals; and *μ*, *ν* for the atomic basis sets. In DFT, the total-energy functional is given as

$$E_{\mathrm{KS}}=T_{s}\left[n\right]+E_{\mathrm{ext}}\left[n\right]+E_{H}\left[n\right]+E_{\mathrm{xc}}\left[n\right]+E_{nuc-nuc}.$$ (1)

Here, *n*(**r**) is the electron density and *T*_s_ is the kinetic energy of non-interacting electrons, while *E*_ext_ is external energy stemming from the electron-nuclear attraction, *E*_H_ is the Hartree energy, *E*_xc_ is the exchange-correlation energy, and *E*_nuc-nuc_ is the nucleus–nucleus repulsion energy. The ground state electron density *n*_0_(**r**) (and the associated ground state total energy) is obtained by variationally minimizing Eq. 1 under the constraint that the number of electrons *N*_*e*_ is conserved. This yields the chemical potential *μ* = *δE*_*KS*_/*δn* of the electrons and the Kohn–Sham single particle equations,

$${\hat{h}}_{KS}\psi_{p}=\left[{\hat{t}}_{s}+v_{\mathrm{ext}}\left(r\right)+v_{H}+v_{xc}\right]\psi_{p}=\epsilon_{p}\psi_{p}$$ (2)

for the Kohn–Sham Hamiltonian ${\hat{h}}_{KS}$ . In Eq. 2, ${\hat{t}}_{s}$ denotes the kinetic energy operator; *v*_ext_ is the external potential; *v*_*H*_ is the Hartree potential; and *v*_*xc*_ is the exchange-correlation potential. Solving Eq. 2 yields the Kohn–Sham single particle states *ψ*_*p*_ and their eigen energies *ϵ*_*p*_. The single-particle states determine the electron density via

$$n\left(r\right)=\underset{i}{\sum }f_{i}|\psi_{i}{|}^{2},$$ (3)

in which *f*_*i*_ denotes the Fermi–Dirac distribution function.

To solve Eq. 2 in numerical implementations, the Kohn–Sham states are expanded into a finite basis set *χ*_*μ*_(**r**)

$$\psi_{p}\left(r\right)=\underset{\mu }{\sum }C_{\mu p}\;\chi_{\mu }\left(r\right),$$ (4)

with the expansion coefficients *C*_*μp*_. In this basis set, Eq. 2 becomes a generalized eigenvalue problem:

$$\underset{\nu }{\sum }H_{\mu \nu }C_{\nu p}=\epsilon_{p}\underset{\nu }{\sum }S_{\mu \nu }C_{\nu p}.$$ (5)

Using the bra-ket notation ⟨.|.⟩ for the inner product in Hilbert space, *H*_*μν*_ denotes the elements $\left\langle \chi_{\mu }|{\hat{h}}_{KS}|\chi_{\nu }\right\rangle$ of the Hamiltonian matrix, and *S*_*μν*_ denotes the elements ⟨*χ*_*μ*_|*χ*_*ν*_⟩ of the overlap matrix. Using the basis set representation, we get the density matrix for the ground state,

$$P_{\mu \nu }=\underset{i}{\sum }f_{i}C_{\mu i}C_{\nu i}^{*}.$$ (6)

The first step in the DFPT self-consistency cycle is to calculate the response of the density matrix using the given expansion coefficients *C* and *C* ^(1)^.

$$P_{\mu \nu }^{\left(1\right)}=\underset{i}{\sum }f_{i}\left(C_{\mu i}^{\left(1\right)}C_{\nu i}^{*}+C_{\mu i}C_{\nu i}^{\left(1\right)*}\right).$$ (7)

Then, using the density matrix formalization, we get the response of the electronic density,

$$n^{\left(1\right)}\left(r\right)=\underset{\mu ,\nu }{\sum }P_{\mu ,\nu }^{\left(1\right)}\chi_{\mu }\left(r\right)\chi_{\nu }\left(r\right).$$ (8)

Furthermore, we can get the response of the total electrostatic potential with response density,

$$v_{es,tot}^{\left(1\right)}\left(r\right)=\int \frac{n^{\left(1\right)}\left(r\right)}{\left|r-r^{\prime }\right|}dr^{\prime }.$$ (9)

Using the response potential, we get the response of the Kohn–Sham Hamiltonian matrix,

$$H_{\mu \nu }^{\left(1\right)}=\left(\int \chi_{\mu }{\hat{h}}_{\mathrm{KS}}^{\left(1\right)}\chi_{\nu }\left(r\right)dr\right).$$ (10)

Here, ${\hat{h}}_{\mathrm{KS}}^{\left(1\right)}$ is the response of the Hamiltonian operator under the homogeneous external electrical field perturbation with strength **ξ** along coordinate direction J.

$${\hat{h}}_{KS}^{\left(1\right)}=\frac{d\left({\hat{h}}_{KS}+{\hat{h}}_{E}\right)}{d\xi_{J}}=v_{\mathrm{ext}}^{\left(1\right)}+v_{H}^{\left(1\right)}+v_{xc}^{\left(1\right)}-r_{J},$$ (11)

where the response of the total electrostatic potential is $v_{es,tot}^{\left(1\right)}$ discussed in the previous paragraph, and the response of the exchange-correlation potential is $v_{xc}^{\left(1\right)}$ . In the case of the LDA functional, the exchange-correlation energy can be written as *E*_*xc*_ = *∫f*_*xc*_(*n*(**r**))*d* **r**. Evaluating the functional derivative in the latter term yields simply

$$v_{xc}^{\left(1\right)}\left[n\left(r\right)\right]=\frac{\partial^{2}f_{xc}}{\partial n\partial n}n^{\left(1\right)}\left(r\right)=\frac{\partial v_{xc}\left[n\left(r\right)\right]}{\partial n\left(r\right)}n^{\left(1\right)}\left(r\right).$$ (12)

In turn, all these components then allow the Sternheimer equation (Shang et al., 2017; 2018), to be set up, the solution of which allows the response of the expansion coefficients *C* ^(1)^ to be updated. We iteratively restart the DFPT loop until self-consistency is reached, i.e., until the changes in *C* ^(1)^ become smaller than a user-given threshold. The polarizability and dielectric constants are computed in the final steps.

$$\alpha_{IJ}=\frac{\partial \mu_{I}}{\partial \xi_{J}}=\int r_{I}\frac{\partial n\left(r\right)}{\partial \xi_{J}}dr.$$ (13)

### 2.2 OpenCL

Open Computing Language (OpenCL) is a unified parallel programming framework for heterogeneous processing platforms, taking full advantage of CPUs, GPUs, and other computing devices. The OpenCL architecture is abstracted into platform models, memory models, and execution models.

The platform model consists of a host connected to one or more OpenCL devices. More specifically, each OpenCL device consists of one or more compute units (CUs) that further consist of one or more processing elements (PEs). An OpenCL application is divided into host code and device code. The host code runs on the host and submits device code to OpenCL devices that execute the computation of device code on processing elements.

The execution model is defined in terms of two units of execution, that is, kernels and a host program. The former executes on one or more OpenCL devices, while the latter executes on the host platform. Before a kernel is submitted for execution, an index space, NDRange, is defined. Each point in NDRange is called a work-item, which executes kernel functions on PE. Work-items are assembled into work-groups allocated to CUs for execution.

The memory model includes two fundamental memory regions: host memory and device memory. Host memory is exclusively available for direct access by the host. Device memory consists of global memory, constant memory, local memory, and private memory. Of these, global/constant memory is shared between the host and device; local memory is shared within a work-group on the CU; and private memory is private to the work-item.

### 2.3 Hygon GPU

A GPGPU is a graphics processing unit (GPU) processor used for purposes other than rendering graphics. A GPGPU has powerful parallel computing ability and plays an important role in scientific computing, AI, cryptography, and other fields. NVIDIA and AMD GPUs are the most popular GPGPUs.

Similar to AMD GCN devices, a Hygon GPU is equipped with 64 compute units, an L2 cache, and 16 GB of global memory. Each compute unit is composed of one scalar unit and four-vector (SIMD) units, while each SIMD consists of 16 vector arithmetic logic units (VALUs) representing 16 processing elements. on the on-chip memory for each compute unit includes 64 KB local data share (LDS), 16 KB of L1 cache, and a 64 KB vector general-purpose registers (VGPRs) file of 32-bit registers located on each SIMD.

Work-groups allocated to a single compute unit are divided into as many as 40 wavefronts following the mapping based on a linear order of work-items. For each SIMD, 16 processing elements allow a wavefront with up to 64 work-items running in parallel. In terms of memory usage, a wavefront can utilize up to 256 VGPRs for each work-item; a work-group can utilize up to a maximum of 32 KB memory of 64 KB LDS.

## 3 Implementation overview

We implemented and accelerated DFPT by first rewriting the three time-consuming simulation stages of the FHI-aims package in OpenCL and then performing a set of optimizations targeting GPGPUs.

**FIGURE 1:**
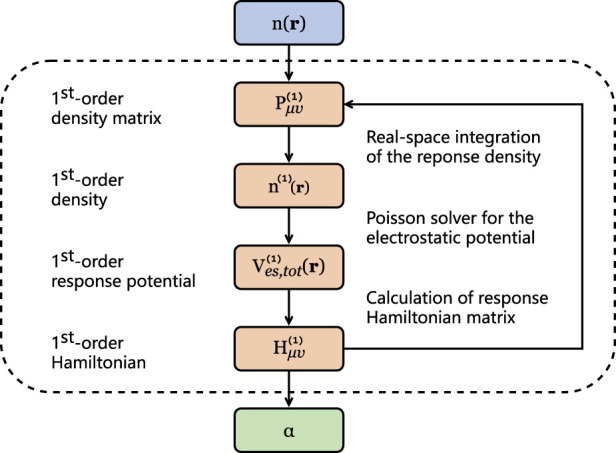
Flowchart for the calculation of density-functional perturbation theory.

When implementing the DFPT work-flow in Figure 1, there exist three extremely time-consuming stages in FHI-aims, as annotated: the real-space integration of the response density (denoted as rho); the Poisson solver for the electrostatic potential (denoted as sum_up); and the calculation of the response Hamiltonian matrix (denoted as H).

**FIGURE 2:**
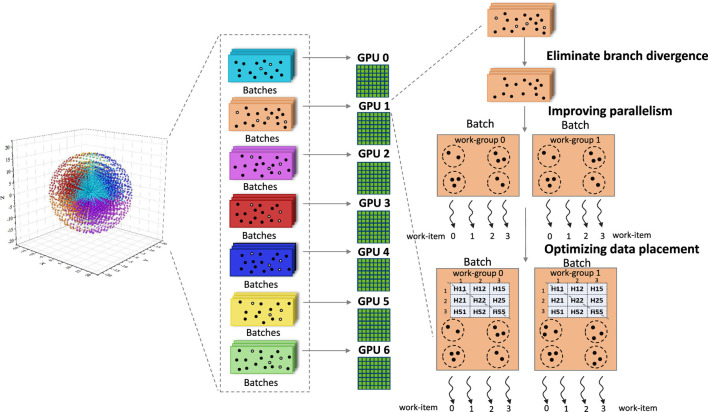
Implementation of FHI-aims.

As shown in Figure 2, FHI-aims first generated a set of grid points that are non-uniform radial spherical (Blum et al., 2009; Delley, 1996; Baker et al., 1994) for more accurate numerical integration. Those grid points were further partitioned into batches following Havu et al. (2009), with each batch containing 100–300 grid points. A set of batches was assigned to an accelerator for processing; typically, each GPGPU would be responsible for hundreds of batches. To improve simulation efficiency, we first eliminated branch divergences by excluding grid points located far from all nuclei before they were sent to the GPGPUs. After that, we improved parallelism from hundreds to tens of thousands by making batches implicit and directly mapping grid points to threads. At the same time, we minimized data transfer among various memory regions to improve memory efficiency by letting frequently used data reside in on-chip memories and trading computations for storage.

## 4 Optimizations on the Hygon GPU

This section states the architectural challenges for application optimizations on the Hygon GPU and then suggests three effective optimizations: eliminating branch divergence, improving parallelism, and optimizing data placement.

### 4.1 Challenges

In general, there exist three main challenges for application optimizations on a Hygon GPU that have been introduced by its micro-architectural features: abundant parallelism, careful data placement, and convergent control flow.

• Abundant parallelism. First, applications are required to provide abundant parallelism to hide long memory access latencies. Once the pipelines of a group (wavefront) of threads are stalled waiting for registers to be ready, they would be swapped out by the GPU hardware. In this case, the application must provide sufficient parallelism so that another group (wavefront) of threads is ready to be swapped in to keep the pipelines busy. As a result, applications that lack parallelism would lead to frequent pipeline stalls and ultimately unsatisfactory performance.
• Careful data placement. Second, applications are required to carefully place data in various types of on-chip and off-chip memory to exploit the complex memory hierarchy. Typically, GPUs contain various types of memories with different accessing modes and delays; for example, global memory that can be accessed by all threads with long latency, shared memory that can be accessed by a group of threads with less delay but limited capacity, and registers that are private to a single thread with minimal latency. Furthermore, data movements between different types of memory would lead to various forms of overhead. As a result, it is non-trivial to determine where to place the data because it has a huge impact on program performance in terms of data locality and inter-thread communication.
• Convergent control flow. Third, applications are required to converge their control flow to avoid wasting computational resources on useless work. GPUs perform computations following the same instruction multiple threads (SIMT) execution model, in which the resolution of control-flow divergence is delayed until the point when computed results must be stored in the main memory. In this case, once a control-flow path has been taken by a thread, it would be also executed in other threads, even if their results were discarded. That is, control-flow divergence would lead to a waste of computational resources doing useless work, which can greatly reduce the performance of the application.

### 4.2 Improving parallelism

As noted in Section 3, FHI-aims exposes multi-granularity parallelism that resides between several sets of batches, several batches, and several grid points in a batch.

Typically, FHI-aims would assign hundreds of batches to each GPGPU, with each batch containing hundreds of grid points. Therefore, fine-grained parallelism, i.e., among grid points, must be exposed for effective GPGPU execution, which generally favors 10^4^ threads or more. We adopted two different strategies to improve the parallelism in the three time-consuming stages based on their data access patterns.

**FIGURE 3:**
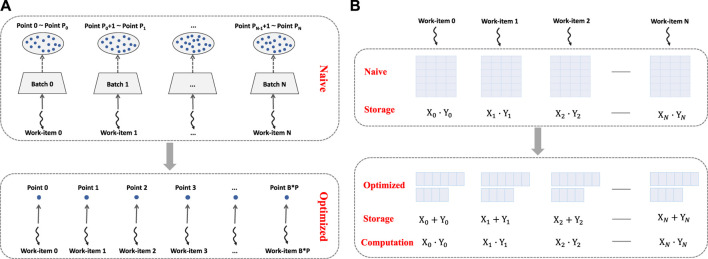
Methods used to improve parallelism. **(A)** Making batches implicit. **(B)** Reducing storage requirements.

For sum_up, there are no inter-grid-point shared data, which indicates that all grid points could be processed independently. Therefore, we first make batches *implicit*, allowing grid points to be mapped onto OpenCL work-items, and allowing an individual batch to be processed by different OpenCL work-groups, as shown in Figure 3A. As a result, the fine-grained parallelism in FHI-aims has been exposed to its upper limit.

However, it is not trivial to achieve this upper-limit parallelism by simply making batches *implicit*. This is because memory capacity in each region would be exceeded with an increased number of work-items or an extended scale of the system, making further parallelism improvement impossible, making the upper limit unreachable, or causing speed to drop. As shown in Figure 3B, in *sum_up*, each thread uses a temporal matrix with a space complexity of *O*(*n* ^2^) to keep its intermediate results, for example, it requires 1 KB storage for each work-item in case SI2. With an enlarged *local*_*size* or *n*, the matrix will overflow from the on-chip cache to the off-chip global memory, leading to a speed drop. To handle this, we choose to keep two vectors in memory, that is, the *coord*_*c*_ and the *Fp*, and delay the calculation of *coord*_*mat* and *rest*_*mat* until the program point at which their values are used. In this way, the space complexity requirements of each work-item would be reduced from *O*(*n* ^2^) to *O*(*n*), enabling *local*_*size* to be increased and relevant data to be moved to private memory in Section 4.4 for further performance improvement.

Alternatively, for rho and H, which read and write the Hamiltonian matrix, respectively, grid points could not be processed fully independently because grid points belonging to the same batch would access the same elements in the matrix. In this case, we first made batches *explicit* and mapped each of them to an OpenCL work-group, and then tried to maximize *local*_*size* (i.e., 256 on the Hygon GPU), making each work-group expose as much parallelism as possible.

### 4.3 Eliminating branch divergence

In general, two types of major control flows exist that could be eliminated in FHI-aims, *static* or *dynamic*.

#### 4.3.1 Static branch divergence elimination

We eliminated some kernel branches by hoisting them to CPU and simplifying the control flows.

**FIGURE 4:**
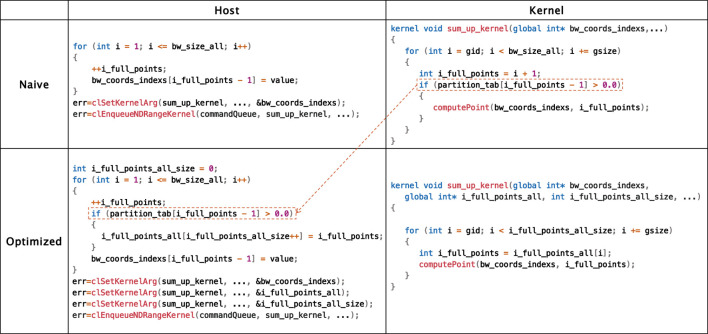
Pseudocode for static elimination of branch divergence.

Hoisting kernel branches to the host. Some grid points that are outside a certain distance threshold of any nucleus should be excluded from processing. Originally, this was determined in the loop iterations over all grid points in a batch; thus, a major branch exists in the OpenCL kernel, as shown in Figure 4. We hoisted this branch from the kernel to the host so that the kernel could load only valid grid points for further processing. As a result, this type of branch could be handled *statically* with the help of OpenCL host codes, leading to convergent control flows in the kernel for effective GPGPU execution.

**FIGURE 5:**
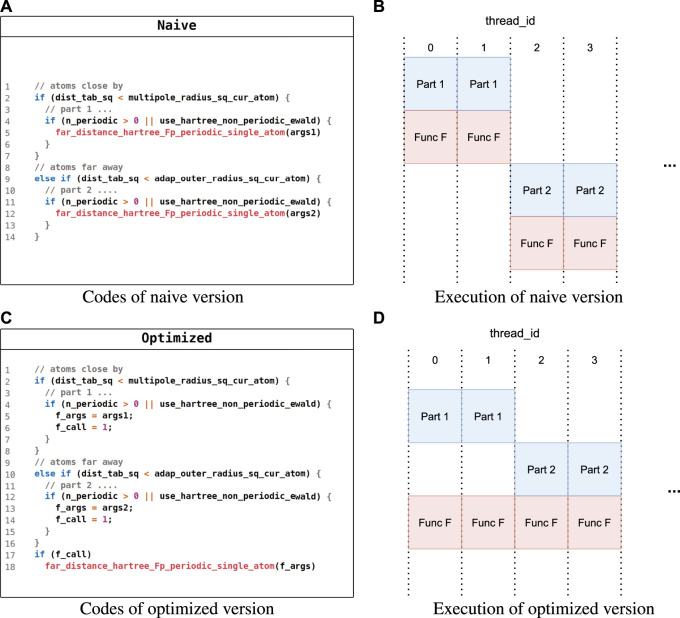
Pseudocode for simplifying kernel control flows. **(A)** Code of the naive version. **(B)** Execution of the naive version. **(C)** Code of the optimized version. **(D)** Execution of the optimized version.

Simplifying kernel control flows. In FHI-aims, there may exist some identical operations in different paths of a complex control flow, and Figure 5 gives such an example in sum_up. In the naive implementation shown in Figure 5A, function far_distance_hartree_fp_periodic_single_atom (function F) could be called in both paths with different arguments, regardless of whether Hartree potential components came from closed atoms or far away atoms. However, function F could not be executed with all SIMD lanes fully exploited if atoms are closed in some lanes but not in others, as shown in Figure 5B. To deal with this, we simplified the control flow by merging identical operations from different paths to make function F called in the same path independent of the distance of the atoms, as shown in Figure 5C. As a result, all SIMD lanes could be fully exploited when executing function F, as shown in Figure 5D.

#### 4.3.2 Dynamic branch divergence elimination

**FIGURE 6:**
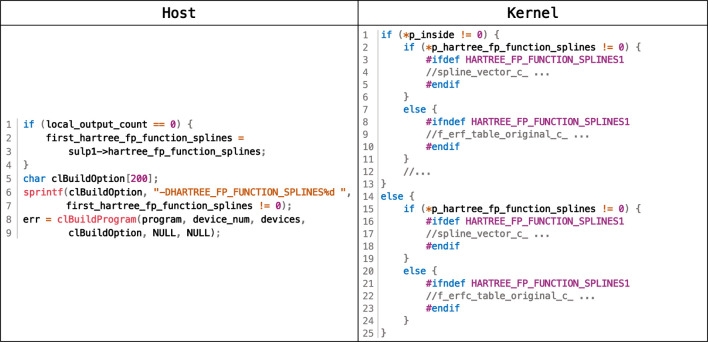
Pseudocode for dynamic elimination of branch divergence.

Some operations are carried out only for some types. This indicates that, given an input, a certain path in the control flow would not be taken for all grid points; thus, the entire path could be eliminated in this case. However, it is not trivial to do this because different inputs require different paths, and furthermore, this is not statically deterministic. We delayed this specialization to the runtime of the OpenCL host code, as shown in Figure 6, eliminating this type of branch divergence *dynamically*.

To be specific, we leveraged the runtime compilation scheme in OpenCL to achieve this. OpenCL embeds the compilation of kernel codes in its host code by invoking the clBuildProgram API, as shown in line 8 of the host code. Compiler options are allowed to be passed via this API to compile kernel codes. Based on this, we first specified whether a control-flow path would be taken for a certain type of input by annotating the OpenCL kernel codes with macros, as shown in the kernel code in Figure 6. After that, we inserted codes in the OpenCL host code to obtain the current input type when executing, as shown in lines 1–4 of the host code. Finally, we applied compiler options indicating the obtained input type to clBuildProgram, as shown in -DHARTREE_FP_FUNCTION_SPLINES at line 6 of the host code. This instructs the compiler to ignore the entire path at lines 7–11 or lines 20–24.

### 4.4 Data placement

Abundant data elements were accessed in FHI-aims, and we placed them into various OpenCL memory regions based on their access pattern to minimize data movement between different levels of the hierarchical memory in the Hygon GPU.

In particular, OpenCL partitions the device memory into four distinct memory regions.

1. Global memory. Data elements can be placed into this memory region using the *__global* qualifier*.* The memory region can be accessed (read and written) by all work-items in the kernel and by the host.
2. Constant memory. Data elements can be placed into this memory region using the *__const* qualifier*.* The memory region is a part of global memory used to store constant variables; that is, it can be read by all work-items in the kernel and by the host.
3. Local memory. Data elements can be placed into this memory region using the *__local* qualifier*.* The memory region is shared by all work-items in a work-group and cannot be read or written by work-items in other work-groups or by the host.
4. Private memory. Data elements can be placed into this memory region using the *__private* qualifier*.* The memory region is private to a work-item and cannot be read or written by other work-items or by the host.

On the Hygon GPU, both *global* memory and *constant* memory are mapped to off-chip DRAM, with *constant* memory cached on the chip and *global* memory not cached. As a result, for multiple-accessed data, the access latency could be significantly reduced if the data residing in *constant* memory were compared with *global memory* if it has been cached. Alternatively, the *local* memory is mapped to LDS, that is, on-chip storage that could be accessed much faster than off-chip DRAM. In particular, LDS has similar latency when accessed as a cache, but LDS must be explicitly managed by the application. The *private* memory is mapped to the vector general-purpose registers (VGPRs) file, which has the lowest memory access latency and smallest capacity.

We effectively exploited the on-chip memory by choosing appropriate data to be placed into *constant/local/private* memory to reduce the data traffic from/to off-chip memory. In general, data that were highly reused were considered candidates for placement, and the placement strategy was determined based on how the data were *shared* and *accessed*.

In particular, the data placement problem extensively exists in modern processors because they typically adopt a multi-level memory hierarchy, with those levels varying in both capacity and latency. Thus, determining into which levels data should be placed is a common and important decision for all applications, including DFPT and DFT. Therefore, the following principles of optimization could be applied to a set of applications for a set of processors.

#### 4.4.1 Constant memory data placement

For *constant* memory, we chose data using the following three criteria: first, the data should be read-only because it is illegal to update elements residing in *constant* memory; second, the data should be shared by all work-items in the same work-group because *constant* memory is cached in the private cache of each compute unit, on which the entire work-group is executed; third, the data should be reused with small reuse distances to avoid being evicted from the cache. In particular, reuse distance is defined as the number of distinct data elements accessed between two consecutive references to the same element (Ding and Zhong, 2003), indicating that data with a small reuse distance could reside in the cache as long as it is alive. For example, the array index_cc was placed onto *constant* memory by suffixing its declaration with *__constant* in sum_up.

#### 4.4.2 Local memory data placement

**FIGURE 7:**
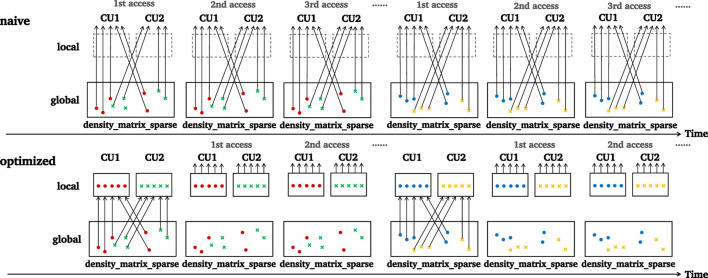
Preloading data from global memory to local memory.

For *local* memory, we chose highly reused data that were shared by all work-items in the same work-group, with no restrictions on their reuse distance. That is because *local* memory is mapped onto LDS, which is managed explicitly by the application and thus would not evict any data element implicitly. Figure 7 shows an example in rho, with each work-group accessing some discrete data elements of the Hamiltonian sparse matrix first_order_density_matrix_sparse , and those data elements are selected as subscripts determined by the matrix dense_from_sparse . We placed the accessed data elements of first_order_density_matrix_sparse into *local* memory because they are highly reused but difficult to keep residing in the cache due to the longer reuse distance. Typically, a work-group would access data elements up to 3.32 MB on Si2, which exceeds the capacity of the LDS. Therefore, we further performed loop tiling to handle this with a tile size of 32. In particular, in each tile, we let each work-item fetch a set of distinct data elements to avoid bank conflict in the LDS and make the fetched data elements visible to all work-items in the work-group by inserting a work-group barrier.

**FIGURE 8:**
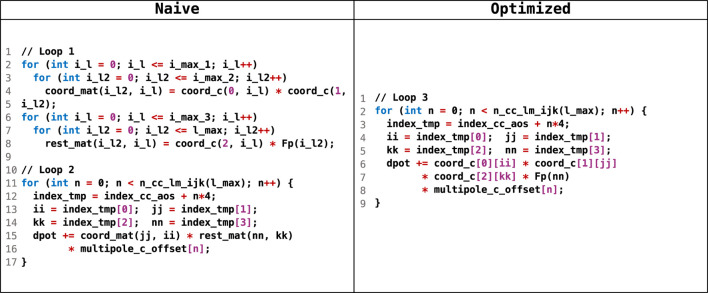
Pseudocode for loop fusion and memory usage reduction.

We also utilized two optimizations to further reduce the data traffic to/from *local* memory. First, we let data shared across various kernel launches reside in *local* memory by allocating them as kernel arguments with a *__local* qualifier. Second, we fused several loop nests to reduce intermediate data, and Figure 8 shows an example from sum_up. In the naive implementation, a two-dimensional array coord_mat and rest_mat was used to keep intermediate results between loops Loop1 and Loop2 , consuming up to 20.25 KB of LDS capacity. After optimization, loops Loop1 and Loop2 were fused, making it sufficient to use the one-dimensional array coord_c to keep intermediate results; thus, the LDS capacity consumption was reduced to no more than 6.75 KB.

#### 4.4.3 Private memory data placement

For private memory, we chose highly reused data that were private to each work-item under its capacity constraint. In particular, if the chosen data element is an element of an array, its subscript should be statically known. For example, we placed the two arrays, wavei and wavej in sum_up, into *private* memory by declaring them with the qualifier *__private*. Accesses to wavei and wavej were enclosed in a two-depth nested loop, with each element of wavei reused in the inner loop and wavej reused in the outer loop. Therefore, putting them in *private* memory could significantly reduce the amount of data taken out of registers.

### 4.5 Portability among various HPC systems

Our OpenCL implementation (Section 3) and its proposed optimizations (Sections 4.2 – 4.4) are easily portable among various HPC systems.

Functional portability is achieved by re-writing FHI-aims with the OpenCL programming interface, which is a cross-platform unified framework supported on a large set of accelerators, for example, Nvidia GPU, AMD GPU, and SW39010. Therefore, it could be executed across various HPC systems and yield correct simulation results without any extra effort.

Performance portability could be expected among various GPUs because our optimizations are designed based on typical GPU architectures. In addition, accelerators with specific target architectures (e.g., software-managed on-chip memory) could also benefit from a part of those optimizations (e.g., the memory placement strategies described in Section 4.4), but only with some detailed parameters tuned (e.g., the size of its on-chip memory). We have included some brief but in-depth analyses in the following.

First, parallelism optimizations can improve performance on accelerators that equip a large set of fine-grained parallel to compute units, with parallelism improved and fine-grained load balancing. This architecture is typical of modern GPUs; the Nvidia GPU has CUDA cores, while the AMD GPU has computing units that yield significant performance benefits by not allowing their compute units to be idle. However, this may not be profitable on processors exploiting a few coarse-grained parallels compute units, such as the A64FX (ARM64 on Fukagu), which includes 48 cores.

Second, control flow optimizations can improve performance on accelerators with parallel compute units that work in a single instruction multiple data (SIMD) or SIMT way, which are common on modern GPUs. In such architectures, severe performance penalties occur when two parallel compute units must execute different instructions, i.e., control flow divergence. However, this may not be profitable on multiple instructions, and multiple data (MIMD) processors (e.g., SW39010) because each compute unit has its PC for execution.

Third, memory optimizations apply to a large set of accelerators, especially those with software-managed on-chip memories. Modern architectures feature a multiple-level memory system, including a set of memories with various latencies and capacities, for example, most-fast-but-rare registers and fast on-chip caches. A common principle of memory optimization that was implemented in our work is to minimize cross-level data movements. Based on this principle, when ported to other accelerators, performance profits could be expected with just a few tunings.

## 5 Evaluation

We evaluated our proposed OpenCL-accelerated FHI-aims on a Sugon supercomputer equipped with Hygon GPUs as heterogeneous accelerators. In particular, each node on the Sugon consists of one Hygon C86 7185 processor and four Hygon GPUs, and the Hygon C86 7185 processor has 32 CPU cores, with each running at 2.00 GHz and connected with 128 GB of memory. FHI-aims is compiled using GCC 7.3 and profiled using AMD rcprof.

### 5.1 Test case information

**FIGURE 9:**
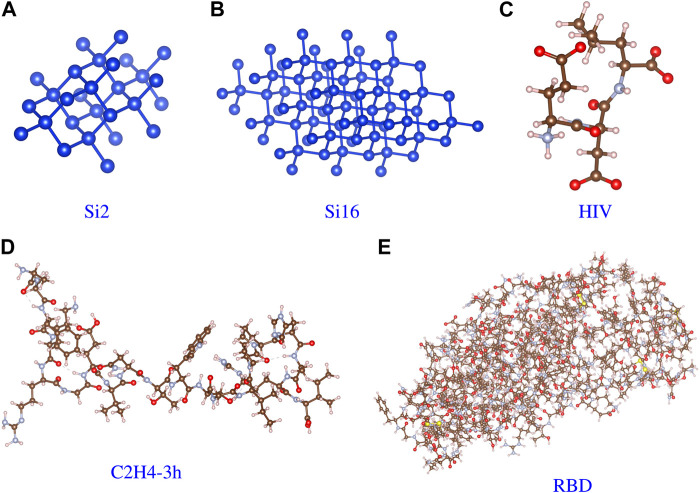
Molecular structure of the test cases. **(A)** Si2. **(B)** Si16. **(C)** HIV. **(D)** Polypeptide. **(E)** RBD.

Table 1 lists some information about the test cases used in our evaluation. In this paper, we focus on the performance of the Hygon GPU on a Sugon supercomputer that is equipped with a 32-core CPU and four GPUs for each node. We performed evaluations across various materials shown in Figure 9, including a crystal system such as a Si atomcase and molecule systems such as the HIV (ligand for HIV-1), polypeptide (C_100_H_144_N_31_O_26_), and RBD (receptor-binding domain on the spike protein of SARS-CoV-2) cases. The number of MPI tasks may be limited by the scale of the case, so we adjusted it to match the case.

**TABLE 1:** Case information and errors.

Case name		Grid	n_atoms	n_basis	n_centers	max batch size	MPI task	GPU(s)	Error
Si-2	#1	56,860	2	50	4394	113	1	1	5.22e−13
	#2	35,836	2	36	4394	72	1	1	3.12e−13
	#3	35,836	2	72	4394	72	1	1	7.08e−13
	#4	35,836	2	36	4394	142	1	1	5.69e−13
	#5	35,836	2	36	4394	282	1	1	6.74e−14
Si-16	#1	454,880	16	144	5488	114	1	1	4.50e−14
	#2	286,688	16	144	5488	73	1	1	9.99e−14
	#3	286,688	16	288	5488	73	1	1	7.23e−14
	#4	286,688	16	288	5488	143	1	1	1.17e−13
	#5	286,688	16	288	5488	283	1	1	1.89e−12
HIV		265,842	49	1,359	49	132	1	1	5.83e−13
Polypeptide		1,673,454	312	940	312	105	8	1	3.21e−11
RBD		16,182,074	3,006	9210	3,006	103	32	4	1.36e−11

### 5.2 Simulation validation

As shown in the rightmost column of Table 1, we obtained almost identical results for the simulations compared to the simulations conducted on the original Fortran version of FHI-aims running on x86 CPUs. The errors were the L2 distances of the DFPT result vectors (DFPT for polarizability or dielectric_constant). The absolute errors of Si, HIV, polypeptide, and RBD were no more than 2e−11 on the Hygon GPU compared with the x86 CPUs. Our results were in good agreement with machine precision, illustrating that realistic problems of scientific investigation can be handled correctly by using GPUs.

### 5.3 Overall performance

**FIGURE 10:**
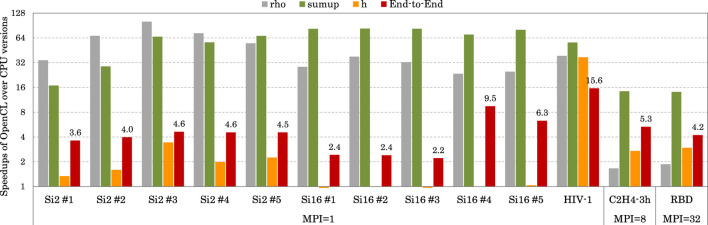
Speedup of the OpenCL version over the CPU version. When computed with one MPI process, the two versions use one core and one GPU, and one CPU core, respectively. When computed with eight MPI processes, the two versions use eight cores and one GPU, and eight CPU cores, respectively. When computed with 32 MPI processes, the two versions use 32 cores and four GPUs and 32 cores, respectively.

Figure 10 illustrates the overall speedup using the Hygon GPU across all test cases. Results show that, compared with using only CPU cores in each node, FHI-aims could be significantly end-to-end accelerated utilizing the Hygon GPU.

For small systems using one MPI process, our OpenCL version achieved end-to-end speedups of up to 15.6× by utilizing one Hygon GPU together with one CPU core compared to using only one CPU core. Among the three time-consuming stages, rho and sum_up were significantly accelerated by 23.4–100.6× and 16.9–82.9×. In comparison, the speedups on H were less impressive (up to 3.4×) because our support for H in DFPT dielectric was not comprehensive.

For a medium system using eight MPI processes, our OpenCL version achieved an end-to-end speedup of 5.3× by utilizing one Hygon GPU together with eight CPU cores, compared to using only eight CPU cores. In particular, the three time-consuming stages of rho, sum_up, and H were accelerated by 1.7×, 14.4×, and 2.7×, respectively, with the time percentages changing from 10.8%/74.8%/14.4%–38.2%/30.6%/31.2%.

For a large system using 32 MPI processes, our OpenCL version achieved an end-to-end speedup of 4.2× by utilizing four Hygon GPUs together with 32 CPU cores, compared to using only 32 CPU cores. In particular, the three time-consuming stages of rho, sum_up, and H were accelerated by 1.9×, 14.1×, and 3.0×, respectively, with the time percentages changing from 7.6%/84.1%/8.3%–31.7%/46.4%/21.8%.

In addition, their energy efficiencies were also analyzed, with the 32-core CPU consuming 180 W and the GPU consuming 300 W. We noted that up to 2.66× of energy-efficiency improvement was obtained by using GPUs to calculate sum_up. However, end-to-end energy efficiencies are not currently satisfactory due to relatively low speedups on rho and H, inspiring us to investigate further optimizations.

### 5.4 Performance breakdown

**FIGURE 11:**
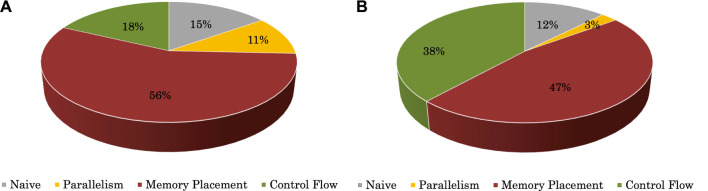
Performance breakdown for sum_up. **(A)** Polypeptide. **(B)** RBD.

Figure 11 shows the performance breakdown for the most time-consuming stage, that is, sum_up, compared with the CPU version. In the figure, relative speedups of the OpenCL version over the x86 version are given, with “Naive” representing a baseline OpenCL implementation described in Section 3, and Parallelism, Control Flow, and Memory Placement denoting three optimizations introduced in Sections 4.2 – 4.4, respectively. Figure 11A shows the case of polypeptide, for which we first applied a baseline OpenCL implementation and contributedto15% of the total speedup of 14.4× (with the CPU version executing for 16.61 s and the baseline OpenCL implementation executing for 4.89 s). Based on this naive OpenCL implementation, we performed three further types of optimizations, including improving parallelism, optimizing data placement, and simplifying control flows, contributing to 11%, 56%, and 18% of total speedup (with execution time reduced from the baseline by 4.89 s–4.09 s, 1.69 s, and 1.05 s, respectively). With optimizations in “Memory Placement,” the percentage of the time memory unit stalled in the kernel execution time was reduced from 14.03% to 1.84%. Similarly, Figure 11B shows the case of RBD, for which simply involving the GPU leads to 12% of the total speedup of 14.1× (with the CPU version executing for 276.55 s and the baseline OpenCL implementation executing for 105.33 s), and the three optimizations of parallelism/data placement/control flows contributed 3%, 47%, and 38% of the total speedup (with execution time reduced from the baseline by 105.3 s–93.695 s, 38.62 s, and 19.56 s, respectively).

Performance breakdown results show that, although simply utilizing the GPU could bring some speedups, most performance improvements came from our intensive optimizations. This indicates that significant extra effort is required to exploit the massively parallel computing capabilities of heterogeneous accelerators. Parallelism optimizations showed little contribution because they were used first and did not affect the key factors that restricted performance. However, they provided the possibility for subsequent optimizations. Also, of all the optimizations, data placement, which benefits the memory subsystem, was the most profitable because sum_up is a memory-intensive stage.

#### 5.4.1 Case study

This section examines the data placement optimization for polypeptide to demonstrate how our optimization successfully exploited the hardware resources in a Hygon GPU.

Table 2 lists some events collected by the Radeon Compute Profiler (rcprof) at the sum_up stage, a memory-bound stage, for the naive and optimized OpenCL versions. Results show that we improved the efficiency of the memory subsystem in two ways. First, the data volume of intermediate results was reduced by loop fusion, resulting in notable decreases of FetchSize and WriteSize , which reduced the number of data access requests. Second, the data layouts of frequently reused arrays were reorganized to improve data locality, leading to a significant increase in L1CacheHit , which reduced the latencies of data fetching. By utilizing these optimizations, the compute unit spent less time waiting for data access and was more efficient; it was busy 20% of the time, compared to 8% in the naive version.

**TABLE 2:** Profiling data of sum_up on the case Polypeptide.

Performance counter	Description	Naive	Optimized
VGPRs	Number of general-purpose vector registers used by the kernel	163	102
SGPRs	Number of general-purpose scalar registers used by the kernel	108	108
VALUUtilization	Percentage of active vector ALU threads in a wave	79.22	89.85
VALUBusy	Percentage of GPUTime vector; ALU instructions are processed	8.03	20.73
SALUBusy	Percentage of GPUTime scalar; ALU instructions are processed	3.47	18.39
FetchSize	Total kilobytes fetched from the video memory	47,021,333.38	4,195,676.44
WriteSize	Total kilobytes written to the video memory	44,166,940.47	8,058,666.25
L1CacheHit	Percentage of instructions that hit the data in the L1 cache	22.13	80.15
L2CacheHit	Percentage of instructions that hit the data in the L2 cache	91.14	84.54
MemUnitBusy	Percentage of GPUTime; the memory unit is active	36.33	61.00
MemUnitStalled	Percentage of GPUTime; the memory unit is stalled	14.03	2.85

## 6 Conclusions

In this paper, we proposed an OpenCL implementation for calculating all-electron density-functional perturbation theory (DFPT) in FHI-aims, which allowed all its time-consuming simulation stages to be effectively computed by utilizing different heterogeneous accelerators. In addition, we also performed a variety of GPGPU-targeted optimizations to improve its parallelism, reduce its branch divergence, and exploit its memory efficiency. Evaluations on the Sugon supercomputer showed that notable speedups can be achieved on various materials.

## Data Availability

The original contributions presented in the study are included in the article/Supplementary Material; further inquiries can be directed to the corresponding authors.
