# Corrigendum: OpenCL-accelerated first-principles calculations of all-electron quantum perturbations on HPC resources

**DOI:** 10.3389/fchem.2023.1239854

**Published:** 2023-06-20

**Authors:** Zhikun Wu, Honghui Shang, Yangjun Wu, Zhongcheng Zhang, Ying Liu, Yuyang Zhang, Yucheng Ouyang, Huimin Cui, Xiaobing Feng

**Affiliations:** Institute of Computing Technology, Chinese Academy of Sciences, Beijing, China

**Keywords:** OpenCL, DFPT, GPU, optimization, heterogeneous

In the published article, the **Funding** statement was erroneously omitted. The correct **Funding** statement appears below:

“This work was supported by the National Natural Science Foundation of China (62232015, 62090024, 22003073, T2222026).”

The authors apologize for this error and state that this does not change the scientific conclusions of the article in any way. The original article has been updated.

